# Record-breaking heat days disproportionately influence heat perceptions

**DOI:** 10.1038/s41598-023-41317-9

**Published:** 2023-10-09

**Authors:** Timothy Hyde, Dolores Albarracín

**Affiliations:** University of Pennsylvania, Philadelphia, USA

**Keywords:** Human behaviour, Attribution

## Abstract

From heat waves to hurricanes, tangible weather experiences have been shown to strengthen personal belief in climate change. We ask whether a high temperature day that breaks local heat records—which is a mathematical construct not directly accessible to the senses—has additional impacts on perceptions of worsening heat, above and beyond that of the absolute temperatures. Matching historical heat records to survey data from the United States, we find that each record heat day in a county in 2022 increases perceptions that excessive heat is getting worse, even when controlling for average temperatures, the number of extreme heat days, and demographic factors. Our estimates suggest that exposure to sixteen record heat days predicts roughly the same difference in excessive heat perceptions as between the average Democrat respondent and a political independent. This effect is stronger for populations that are more skeptical of climate science, including Republicans, as well as respondents with weaker beliefs in climate change and more frequent consumption of conservative media. We close with recommendations for media framing of local record-breaking heat events and call for more research on how media outlets cover record-breaking heat.

## Introduction

Even though climate scientists have used counts of record heat days to quantify the degree of climate change in a certain area^1–4^, we know little about the impact of record heat days, which involve a mathematical rather than a sensory determination, on subjective climate perceptions such as the sense that the weather is now hotter than before. However, studies on the relation between weather and beliefs about climate change has found some evidence that personal experience of hot weather, storms, and floods can persuade individuals that climate change is real^5,6^. Therefore, in this paper, we contribute to the literature on weather experience and climate perceptions by isolating an aspect of climate that is not physically tangible but is socially salient: record heat days. We define a record heat day as a day when the high temperature in a locality is higher than any other high temperature on record for that locality and calendar day.

For the purpose of understanding the role of weather experience in climate beliefs, record heat days have three desirable characteristics: (a) they are not physically tangible, (b) they are not directly experienced, and (c) they occur idiosyncratically. When an individual steps outside, they can certainly perceive extreme heat on their skin, but they cannot know whether a hot day is the hottest on record for their area on a particular date unless they have already checked the news. At the same time, record-breaking heat provides a ready-made topic for water-cooler conversations. Meanwhile, a given hot day only becomes a record heat day by happenstance, depending on whether it corresponds to the precise date of other heat waves in previous years. We show that the number of record heat days in a county is minimally correlated with other important cues for climate change beliefs, including average heat levels, number of extreme heat days, or extreme weather events such as floods, tornadoes, or hurricanes (see “Methods” for this analysis). The question is then: Does the number of record heat days exert an additional impact on climate beliefs than hot weather alone does not?

## Literature review

The connection between weather experience and climate beliefs has received ample research attention. Researchers have attacked this question from many different angles, examining various types of weather events, multiple measures of experience (subjective vs measured), and a variety of different methodological approaches. A comprehensive meta-analysis of 73 papers found some studies showing evidence of a connection but many others producing null findings, especially among those with methodologies best designed to estimate a causal effect^6^.

A range of credible studies have found that recent exposure to hot weather increases people’s confidence that climate change is occurring and concern about its implications^7–11^. These associations have been found with respect to both objective weather observations and subjective heat perceptions^8^ and across shorter and longer time scales (for the cumulative impact of heat over timescales like months, see^12^). In these studies, heat exposure is typically defined as either (a) temperature measured on the day of the belief report, (b) subjective evaluations of recent heat, or (c) abnormally high temperatures over a period relative to long-term averages. A subset of these studies have shown that hot weather exerts a stronger influence in populations with greater scope for belief change, notably those that are politically conservative^11,12^.

Other studies have failed to find a significant causal effect of heat on climate beliefs^13–16^. In some cases, the connection disappears when studies introduce a more complete set of controls, suggesting that an already modest association is due to other factors. For instance, controlling for political ideology and personal beliefs about climate change can eliminate the association between recent temperatures and perceived risk from climate change^17^.

A smaller literature has focused on how discussions of particularly hot summer days and natural disasters can make these events socially salient. An analysis of Twitter posts in the U.S. from 2014 to 2016 showed frequent discussions of excessive heat, although conversation volume declined over the course of the two-year study as people seemingly become habituated to hot weather over time, a phenomenon the authors term “declining remarkability”^18^. In a study of Virginia residents, those who report paying closer attention to local TV weather reports were also more likely to believe that extreme weather was becoming more frequent, an association that was strongest for those expressing trust in their local TV meteorologist^19^. However, this analysis did not focus on record heat days specifically, which is our focus.

Despite this important work, the impact of recent record heat days on excessive heat perceptions has not been ascertained. All of these studies have included measures of heat that respondents could physically sense. By contrast, we consider the role that record heat days—a mathematical construct, not physically tangible—may play in driving climate perceptions.

Using survey data from a probability sample of U.S. adults, combined with historical temperature records from the U.S. National Oceanic and Atmospheric Administration (NOAA), we test and find support for the hypothesis that the number of record heat days in a local area during 2022 increases perceptions of excessive heat, above and beyond the effect of overall heat exposure. The survey involves various measures of weather perceptions, including questions about climate change science, storm severity both locally and nationally, and perceptions of various weather events in 2022 compared to previous years. The NOAA data from over 6000 weather stations across the U.S. provide the basis for calculating the record heat days variable for each county as well as both the degree of abnormal heat in 2022 and long-term temperature averages obtained between the years of 1949 and 2021.

Defining perceptions of excessive heat worsening as the answer to the question “To the best of your knowledge, how did excessive daytime heat across the United States in 2022 compare with previous years?”, we find that more record heat days in a county result in more severe perceptions of nationwide excessive heat but do not significantly affect climate change beliefs. The association between record heat days and perceived excessive heat is stronger in populations with greater climate skepticism, namely Republican voters, conservative news viewers, and individuals who do not believe climate change is currently happening. We conclude that nontangible weather events can exert important influences on climate beliefs, presumably mediated by word-of-mouth communication and media reports, and propose further research into this mechanism.

## Results

### Descriptive statistics

Table 1 displays the distribution of the key dependent variables from the survey data (the “Methods” provide a detailed summary of survey mechanics and construction). The reference question for the questions in Panel A was as follows: “To the best of your knowledge, how did [weather concern] across the United States in 2022 compare with previous years?” In every case, respondents perceiving worse weather in 2022 relative to previous years outnumbered those perceiving better weather by at least a factor of 2. In the case of excessive daytime heat, which is the outcome of focus for most analyses in the present study, the ratio is 8. This imbalance is understandable because the contiguous U.S. had its third hottest summer on record in 2022^20^.

**Table 1:** Distribution of climate change beliefs and weather perceptions in survey data (*n* = 1605).

Panel A. Weather in 2022 across U.S. relative to previous years	Much worse	Somewhat worse	About the same	Somewhat better	Much better
Excessive daytime heat	16%	40%	37%	6%	1%
High overnight temps	10%	35%	47%	7%	2%
Drought conditions	23%	37%	32%	7%	2%
Rivers and coastal floods	10%	30%	46%	12%	2%
Hurricanes	9%	30%	43%	15%	3%
Wildfires	13%	30%	40%	15%	3%

Panel B. Climate change	Yes	No	Don't know		
Climate change occurring	74%	12%	14%		
Climate change human-caused (asked only of climate change affirmers)	70%	20%	11%		

Panel C. Disaster trends	More severe	About the same	Less severe		
Weather-related disasters (U.S.)	66%	27%	6%		
Weather-related disasters (your community)	40%	49%	11%		

Panel B in Table 1 details survey responses about climate change. A large majority (74%) affirmed a statement that climate change is occurring, and 70% of those affirmers associated it with human activity. These numbers are largely in line with recent results from the long-running Climate Change in the American Mind survey conducted by the Yale Program on Climate Change Communication^21^, which was the source for our question wording.

Finally, Panel C in Table 1 shows responses to a pair of questions about trends in weather-related disasters. Respondents were asked: “In your opinion, would you say weather-related disasters [around the country/in your community] in the past few years are less severe, more severe, or has there been no change?” Again, more respondents believe disasters are getting worse than believe they are getting better, although they are on average more sanguine about conditions in their own communities than the country at large. Note that the time period cited in this question (“past few years”) does not correspond exactly to our perceptions measure because the question about weather-related disasters appeared in multiple survey waves whereas the perceptions questions about excessive heat and other weather events (see Panel A) were asked only at the tail end of 2022 and specifically referred to that year.

**Figure 1:**
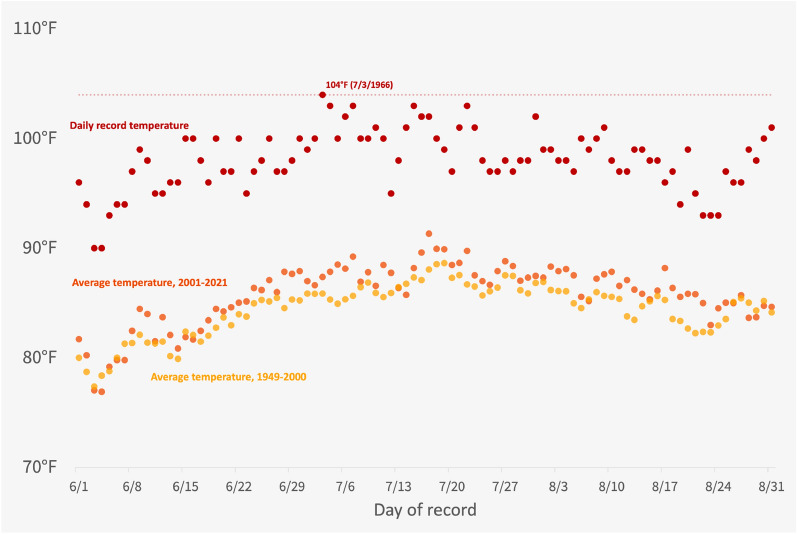
Daily high temperature records for Philadelphia, 1949–2021. Mean and record high temperatures at Philadelphia International Airport for each calendar day in June, July and August, as calculated from records in the Global Historical Climatology Network database.

The “Methods” provide more details on how daily heat records are calculated and determined. Before we report the results, we briefly discuss the distribution of record heat days and argue that they constitute a natural experiment. Figure 1 illustrates the variability of daily heat records. The figure displays average high temperature readings from the weather observation station at Philadelphia International Airport for each calendar day of June, July, and August. These averages are calculated using 73 years of data from 1949 to 2021 inclusive. Two different series are presented: (a) average data from the twentieth century (1949–2000) in yellow and (b) average data from the twenty-first century (2001–2021) in orange. The overall warming trend is apparent.

Figure 1 is useful for clarifying the meaning of single-day and all-time heat records. Record high temperatures for each calendar day appear in red and are the highest recorded high temperature on a particular day during the 73-year span. For example, the single-day record high for June 1 is 96°F. This point contrasts with the all-time high temperature record for this station, which is 104°F, set on July 3, 1966. Therefore, a high temperature of 97°F on a future June 1 would qualify as a single-day heat record for this station, but not an all-time heat record.

Naturally, the average temperature data are much smoother across calendar days than the record-high temperature data. The number of record-breaking heat days in a given county is partly a function of the calendrical alignment or misalignment of present heat waves and past heat waves, and, as such, is a noisy signal of overall heat trends. For example, a hypothetical heat wave with three consecutive days of 100°F heat in Philadelphia would not break any daily heat records if it occurs in the first week of July, but would break three daily records if it occurred any time in the last week of that month. This historical dependency has the effect of decoupling the overall level of excessive heat from the number of record-breaking days.

**Figure 2:**
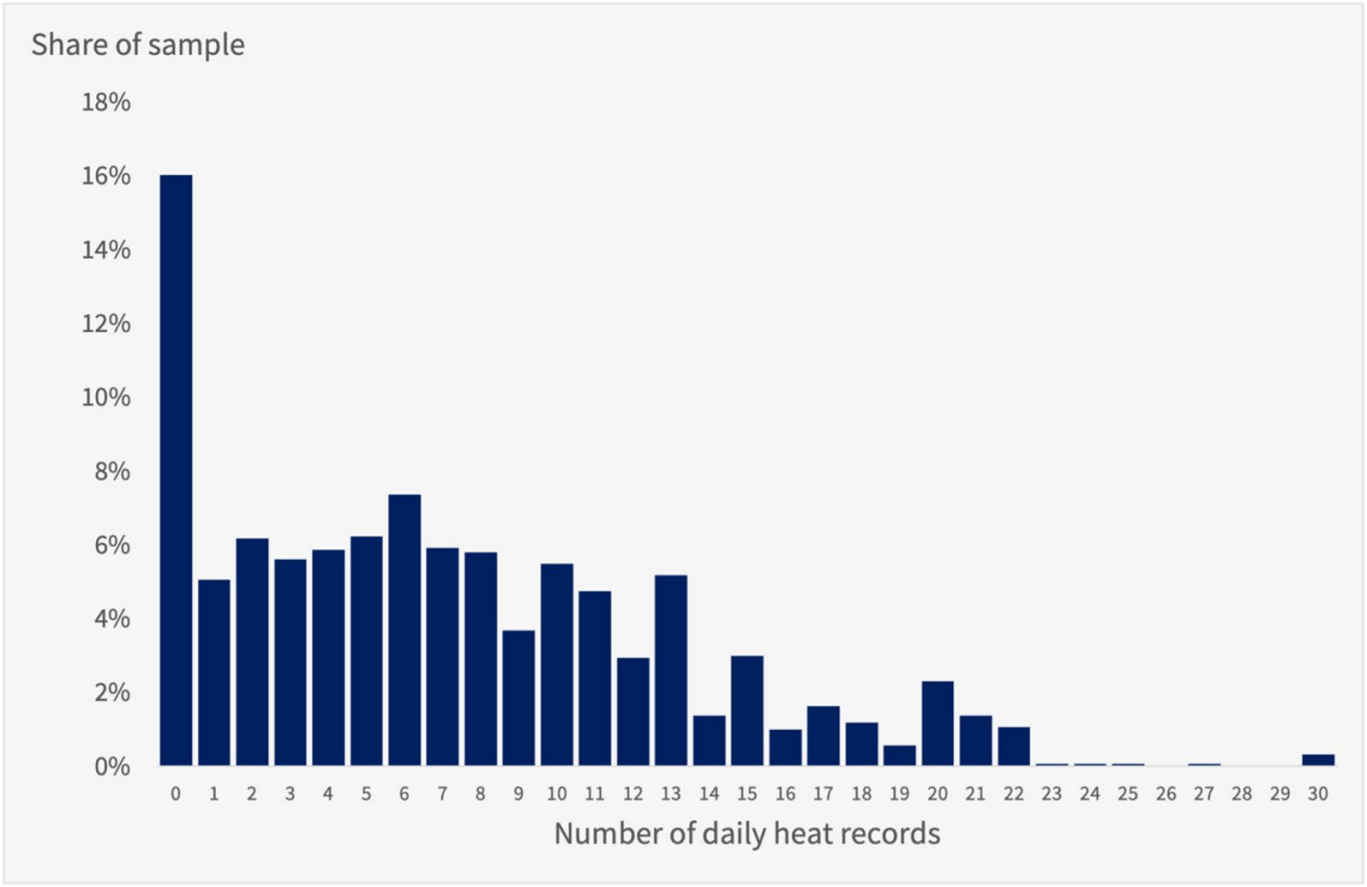
Distribution of record heat days in analytic sample. A histogram of the number of record heat day exposures for each respondent in the analytic sample.

The unpredictable temporal distribution of heat waves across the summer months creates a natural experiment where some counties experience many record-breaking heat days while other counties with similar levels of abnormal heat do not see any records broken (abnormal heat is a measure of the average temperature over a period relative to historical averages; see “Methods” for the definition). Figure 2 displays the distribution of the record heat days variable across the sample. As this quantity only varies at the county level, respondents in the same county are assigned the same value. Table 2 displays the same data, as well as distribution information for the 2022 abnormal heat and historical average high temperature variables. As only 16% of respondents live in counties with no record heat days in 2022, most respondents are exposed to at least one day of record-breaking heat.

**Table 2:** Distribution of heat measures in analytic sample in 2022.

Variables	Mean	Std. dev.	Median	25th %ile	75th %ile	Max value
Abnormal heat	0.14	0.15	0.13	0.04	0.25	0.74
Average high temperature (°F)	68.5	9.4	67.6	60.1	76.9	88.5
Record heat days	7.2	6.0	6	2	11	30
Share with any record	84%					

### Effects of record heat days on excessive heat perceptions

We use ordinary least squares regression models to examine whether exposure to more record-breaking heat days leads to stronger perceptions that heat is worsening over time and beliefs in climate change (see “Methods” for more detail on model specifications). We argue that the residual variability in record heat days conditional on average heat levels constitutes a natural experiment to test the hypothesis that exposure to record heat days causes shifts in excessive heat perceptions and climate change beliefs. Table 3 lists the control variables used in each specification. These controls included a variety of meteorological controls such as long-term average temperature and a disaster acceleration rate described in the “Methods” section.

**Table 3:** Control variables included in regression models.

Meteorological controls	Political controls	Demographic controls
Abnormal temperature 2022 Long-term average high temperature Long-term disaster rate (1953–2022) Disaster acceleration rate (see “Methods”) Indicator for disaster Summer 2022 Indicator for disaster Autumn 2022 County latitude County longitude Days in 2022 with highs above 90°F Days in 2022 with highs above 100°F Days in 2022 with highs above 105°F Days in 2022 with highs above 110°F	Democrat indicator Republican indicator Days/week of national news consumption Days/week of liberal news consumption Days/week of conservative news consumption	Age band fixed effects Gender fixed effects Education level fixed effects Race fixed effects County metro area status indicator Home ownership indicator

Table 4 displays the results of three regression models, a simplified model including only meteorological controls, and two models with additional controls, including our preferred specification with the full set of controls from Table 3 (Column 3). In line with our hypothesis, we find a significant positive coefficient on number of record heat days, indicating that people exposed to more record heat days perceive excessive heat across the U.S. in 2022 as more severe than previous years. The estimated effect is attenuated but still significant when demographic controls are included to account for spatial correlations in the application of the record heat days “treatment.” As expected, Democrat party ID is associated with greater perceptions of excessive heat, as is national news consumption, which we defined as the number of days per week that respondents report watching TV news from a given source. Conservative news consumption is strongly associated with less severe perceptions of excessive heat, even when separately controlling for party ID.

**Table 4:** Effect of record heat days on excessive heat perceptions.

	(1)	(2)	(3)
	Meteorological controls	Meteorological + political controls	Additional demographic controls
# of record heat days	0.0138*** (0.00380)	0.0122*** (0.00356)	0.00843** (0.00354)
Abnormal heat in 2022	0.0937 (0.292)	0.0616 (0.299)	0.111 (0.262)
Average historic temp (°F)	− 0.0191* (0.0104)	− 0.0215** (0.0104)	− 0.0215** (0.00996)
Annual disaster rate	0.0970 (0.116)	0.00746 (0.125)	0.0114 (0.126)
Days above 100°F	0.00158 (0.00262)	0.00158 (0.00248)	0.00162 (0.00243)
Democrat		0.157** (0.0595)	0.133** (0.0565)
Republican		− 0.0499 (0.0605)	− 0.102* (0.0600)
National news consumption		0.0785*** (0.0165)	0.0609*** (0.0198)
Liberal news consumption		0.00896 (0.0129)	0.0152 (0.0138)
Conservative news consumption		− 0.0701*** (0.00853)	− 0.0658*** (0.00985)
Additional demographic controls (see Table 3)			✓
Observations	1605	1605	1605
R-squared	0.027	0.105	0.149

OLS regression models where the dependent variable is excessive heat perceptions on a five-point scale. Greater values of the dependent variable indicate belief that excessive heat is more severe in 2022 relative to past years. Other controls included but not displayed: disaster rate acceleration, indicators for disasters in 2022, county latitude and longitude, counts of days above 90°F, above 105°F, and above 110°F. Additionally included in model in Columns 3 only: age band fixed effects, gender fixed effects, education level fixed effects, race fixed effects, county type (metro vs rural) fixed effects, home ownership indicator. Robust standard errors clustered at the state level in parentheses. Statistical significance indicated as follows: ***p < 0.01, **p < 0.05, *p < 0.1

The coefficient estimate in Column 3 suggests that each additional record heat day a respondent experienced during 2022 caused a movement of 0.008 points up the five-point response scale. At first blush, this seems like a very small effect, but we offer some additional context. First, most respondents are concentrated at points 3 and 4 of the scale, “about the same” and “somewhat worse” respectively, while the average response is 3.6. This implies that even a one-point movement on the scale can shift respondents from a relatively optimistic view (point 3) to a relatively pessimistic view (point 4). Second, as the average respondent was exposed to 7.2 record heat days in 2022, the estimated total effect averages 0.06. Finally, the coefficient on record heat days in Column 3 is approximately one sixteenth as large as the coefficient for Democratic party ID in the same model, suggesting that exposure to sixteen record heat days predicts roughly the same difference in excessive heat perceptions as between a Democrat and a political independent. This is a large number of record heat days for one year but not extremely unusual given that approximately 10% of our sample lived in areas that had 16 record heat days or more in 2022.

### Heterogeneous effects

**Figure 3:**
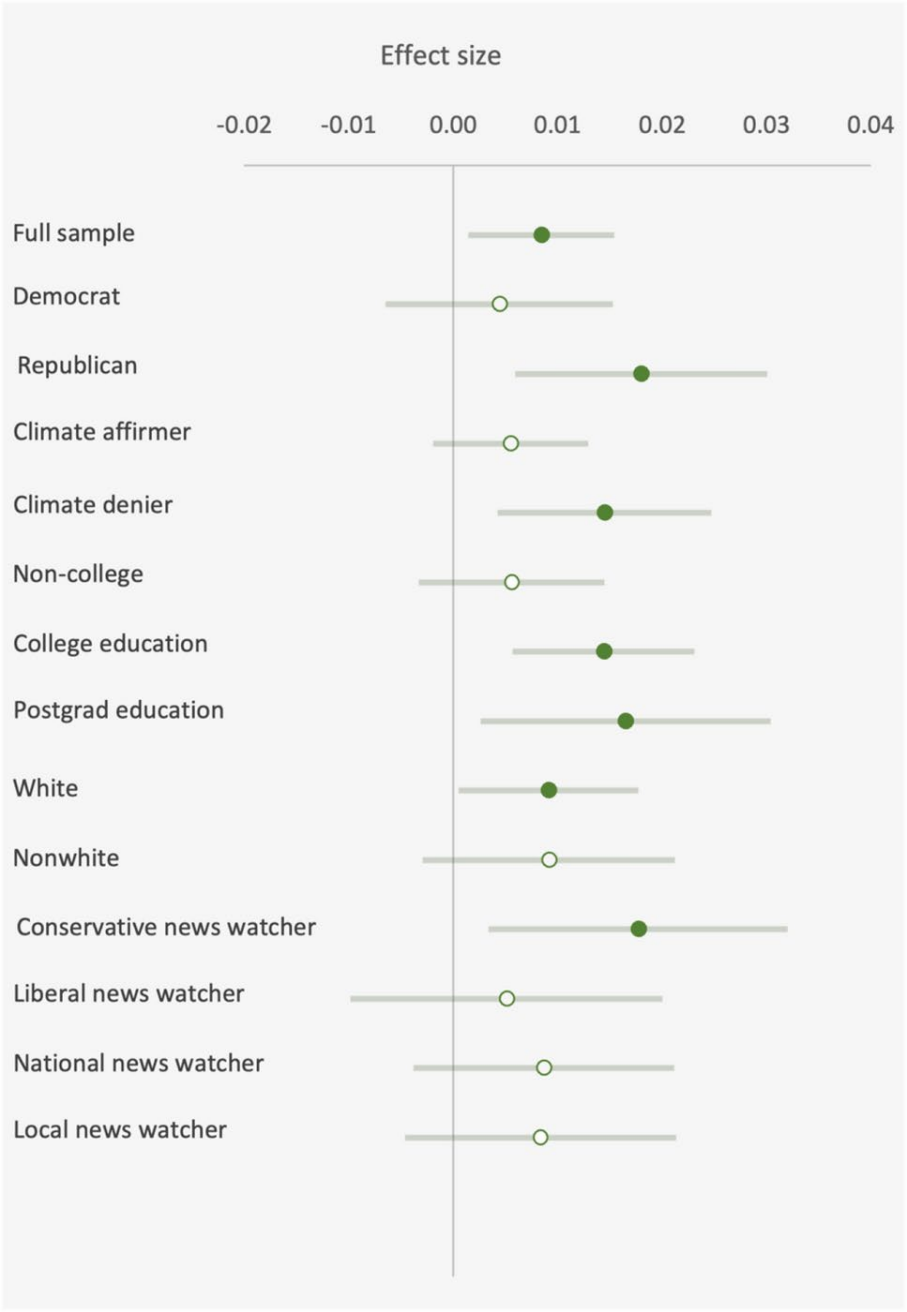
Heterogeneous effects analysis. Point estimates and confidence intervals for *B*_*r*_ from the model in Eq. (2) when estimated on various subsets of the sample. The result for the full sample corresponds to the estimate displayed in Table 1, Column 3.

Figure 3 displays point estimates and 95% confidence intervals for models run on relevant subsamples. As shown, the estimated effect sizes are stronger for Republicans than Democrats, for climate deniers than climate affirmers, and for viewers of conservative TV news than liberal TV news viewers. These effects dovetail well with prior reports that hot weather affects climate beliefs more among populations that are otherwise skeptical about climate change^11,12^. Although the differences in Fig. 3 are not statistically significant, in part because the hypothesis tests are underpowered due to low sample sizes, the correspondence between each group is suggestive and echoes previous findings. Non-college respondents, who are also more likely to be in these skeptical groups, show a smaller effect than college graduates, suggesting that the effect is concentrated among groups that are skeptical but more highly educated. Although the group of college-educated climate skeptics and conservative news viwers is too small in our data for reliable inference, we propose exploring this subgroup with more data, possibly longitudinal, in the future, and presently discuss this finding in relation to the absence of an effect on climate change beliefs (see Discussion section).

### Seasonal effects

We also find that the effect of record heat days on worsening heat perceptions can vary over the course of the year. There are many reasons to think that people would respond differently to records in different seasons. New heat records set during the summer involve the highest absolute temperatures, so they might be the most salient and memorable. Conversely, heat records set during colder times of the year may receive particular attention because they may be perceived as unusual or disconcerting. When we replace the record days variable in our model with a count of records set in a given month and recalculate average temperature and abnormal heat variables at the month level, we find strong positive and statistically significant effects in January, February, April, and September and generally positive but non-significant effects in all other months. These results suggest that records set during colder months may have a larger impact on respondent perceptions, but the inherent noisiness in the data and the fact that we only have perceptions data from a single year warrant caution in interpreting the result. Future work should verify these patterns and examine possible mechanisms. For example, the content of media surrounding heat records may be different at different times of the year.

### Effects on other weather perceptions and climate beliefs

We also run alternative versions of our models with various other dependent variables drawn from the survey data. Table 5 shows that record heat days do not significantly affect perceptions of other weather phenomena beyond excessive daytime heat (Columns 2–7), except for a statistically marginal effect on drought perceptions. These results provide a reassuring falsification test for our main result and reduce concern that spatial correlation in treatment is driving a spurious result.

**Table 5:** Effect of record heat days on perceptions of various weather events.

	(1)	(2)	(3)	(4)	(5)	(6)	(7)	(8)
	Perceptions about 2022 versus prior years (5-point scale)						Weather-related disasters	Climate change belief
	Excessive daytime heat	High overnight temperatures	Drought conditions	River and coastal flooding	Hurricanes	Wildfires		
Record heat days	0.00843** (0.00354)	0.00561 (0.00464)	0.00655* (0.00388)	0.00376 (0.00455)	0.00599 (0.00391)	0.00333 (0.0045)	0.000616 (0.00237)	0.00157 (0.00172)
Meteorological controls	✓	✓	✓	✓	✓	✓	✓	✓
Political controls	✓	✓	✓	✓	✓	✓	✓	✓
Demographic controls	✓	✓	✓	✓	✓	✓	✓	✓
Observations	1605	1605	1605	1605	1605	1605	1605	1605
R-squared	0.149	0.125	0.141	0.128	0.103	0.100	0.257	0.220

OLS regression models where the dependent variable is perceptions of the severity of various weather concerns in 2022 versus prior years on a five-point scale (Columns 1–6), perceptions of weather-related disasters in recent years versus past years on a three-point scale (Column 7). Greater values of the dependent variable indicate worse perceptions of recent weather versus prior weather or in the case of Column 8, belief that climate change is occurring. Column 8 qualifies as a linear probability model, as the dependent variable is dichotomous. Other controls included but not displayed: all those in Table 4, plus disaster rate acceleration, indicators for disasters in 2022, county latitude and longitude, counts of days above 90°F, above 105°F, and above 110°F, age band fixed effects, gender fixed effects, education level fixed effects, race fixed effects, county type (metro vs rural) fixed effects, home ownership indicator. The model displayed in Column 1 is identical to that displayed in Table 1, Column 3. Robust standard errors clustered at the state level in parentheses. Statistical significance indicated as follows: ***p < 0.01, **p < 0.05, *p < 0.1

However, record heat exposure does not lead to statistically significant changes in climate change belief as measured by the question from the Yale Program on Climate Change Communication (Column 8). We speculate that climate change beliefs are less liable to shift in response to record heat days than are perceptions about weather trends because those beliefs are more ideologically motivated and sometimes a core part of one’s political identity^22^. In other words, stating that weather in 2022 is worse than past years is not as politically or ideologically charged as stating a belief in climate science, and is in principle consistent with denial of long-term climate change. It seems reasonable to assume that people are quicker to update perceptions of excessive heat than their self-report of climate belief, especially because the effect of heat days on excessive-heat perceptions is concentrated in climate-skeptical populations (see Fig. 3). Further research is necessary to determine the process by which changes in perceptions eventually do or do not drive changes in beliefs with a strong political resonance.

## Discussion

We find that exposure to record-breaking heat, which is a function of historical data and only loosely tied to overall temperature levels, has a significant effect on perceptions of increases in excessive heat in the United States. This result is in line with our hypothesis that record heat days drive changes in perceptions, even if those events are not tangible nor technically very informative about the degree of excessive heat a region is experiencing.

This finding complements important past research findings about how climate beliefs can be shaped by respondents’ tangible physical experience with oppressive heat, hurricane-force winds, or wildfire smoke. The fact that heat records are not physically tangible suggests that the only possible pathway for these records to influence perceptions is through social interest, weather reports in the media, and conversations about weather. Discussion of record-breaking heat (or record cold, or record precipitation) is typically the only context in which meteorologists or newscasters draw explicit comparisons of current weather to historical climate data. The finding that a few historic outlier temperatures seem to weigh more heavily on the mind that continual above-average temperatures can help inform strategies to persuade people as to the reality and immediacy of climate change.

We were also interested in determining if the effects on heat perceptions translate into beliefs in climate change. One key mechanism is how media outlets handle extreme event attribution (EEA), the science of quantifying the connection between specific weather events and anthropogenic climate change. Media outlets tend to report attribution of specific events differently, partly reflecting scientific uncertainty^23^ but also different ideological commitments. On the one hand, when record-breaking heat is reported, many media outlets could highlight connections with the science of climate change. If the media label record heat as evidence of climate change, record heat days should heighten both perceptions of excessive heat and beliefs in climate change. On the other hand, people with a conservative ideology and a lower educational level are likely to be exposed to conservative media that cover weather events without connecting it to climate change. Moreover, the same media may derogate the science of climate change as part of their regular political content. As a result, record heat may affect heat perceptions but remain disconnected from beliefs in climate change for these populations. Future research examining the actual content of media reports surrounding record heat events can shed more light on which communication strategies seem to do the most to shift beliefs.

## Methods

### Data sources

Survey data are drawn from a longitudinal survey that included questions about recent trends in weather-related disasters both locally and nationally, beliefs about climate change, and the relation between severe weather and climate change. It also included a wealth of demographic variables (e.g., age, race, ethnicity, gender, education level, political party identification, home ownership, and county of residence) and questions about respondents’ media consumption habits. The survey involved a probability sample obtained with a hybrid online/phone methodology, and three waves of the survey were fielded in 2022. The survey design was reviewed and approved for exemption from IRB oversight by the Institutional Review Board at the University of Pennsylvania. We obtained informed consent from all survey participants before recording their responses. The experiment was conducted in accordance with applicable guidelines and regulations.

The key outcome variable of interest in this study is the responses to questions about climate change beliefs and perceptions of weather events in 2022 compared to previous years. Our analyses are cross-sectional because the study measured these perceptions only during the third wave at the end of 2022 (responses gathered in the second and third weeks of December). We limit the analytic sample to respondents who participated in the third wave of the survey, did not have missing data or refused responses for key variables, could be matched to a county of residence, and lived in counties with reliable historical weather information. We additionally exclude one observation from Monroe County, Florida, which had an outlier number of record heat days in 2022 (a total of 129, more than four times any other county in the sample). The resulting sample of 1605 respondents is 91% of the total third wave sample population, and the demographics closely resemble the makeup of the sample at large. The sample includes respondents from 625 different counties across all fifty states and Washington D.C.

Historical temperature data are drawn from the Global Historical Climatology Network maintained by the U.S. National Oceanic and Atmospheric Administration (NOAA). NOAA keeps meticulous records of temperatures, precipitation, and other weather data recorded at more than 6100 observation stations across the United States, going back over 100 years in some cases. We limit our sample to the period from 1949 to present for which data has undergone quality assurance checks and are most easily accessible from the online database. We use daily high temperature readings from this dataset to calculate historical averages at each station. We then average temperature readings across stations in counties with more than one station, excluding a few geographically small rural counties that lack any station.

These NOAA records are also the basis for determining whether a day’s high temperature is record-breaking and qualifies as a record heat day. We obtain data on record heat days by station using a different database compiled by the NOAA National Climatic Data Center, rather than compute them directly using our historical data, to account for records that were originally set before our 1949–2021 data window. To allow comparability between eras with different sensor technology, temperature data are recorded in relatively coarse units (whole degrees Fahrenheit), so there are many occasions when a past record is exactly tied but not exceeded. Only days with records that were broken, rather than merely tied, are counted in our analysis. We tally the number of record-breaking events at each weather station throughout the course of 2022, and aggregate the data to the county level in counties with more than one station by taking the maximum tally across all stations in those counties.

We supplement the above sources with other geographic data to control for county-level variation in climate. In addition to average temperature and abnormal temperature averages calculated from the NOAA weather station data, we collect historical data on presidential disaster declarations by county to obtain a long-term disaster rate for each county. Finally, we include data on each county centroid latitude and longitude obtained from U.S. Census Bureau to control for geographic factors not otherwise captured by the temperature data.

### Variable construction

We follow^12^ in calculating the abnormal temperature fluctuation in each county ${A}_{c}$ as follows:

1 $${A}_{c}=\frac{1}{n}{\sum }_{\mathrm{d }} \frac{{t}_{cd,2022}-\overline{{t }_{cd}}}{{\sigma }_{\mathrm{cm}}},$$

where ${t}_{cd,2022}$ is the high temperature in county *c* on day *d* in 2022, $\overline{{t }_{cd}}$ is the average high temperature reading for county *c* on day *d* throughout the 1949–2021 period, and ${\sigma }_{cm}$ is the standard deviation of high temperatures in county *c* in month *m.* This quantity is summed across all days in 2022 and divided by the number of days with readings *n* to generate an average for each county. The average high temperature for each date $\overline{{t }_{cd}}$ is calculated using a centered seven-day running average to reduce variability. The resulting abnormal heat variable captures continual above-average heat over an extended period rather than just certain extreme days.

Additionally, we define a new variable called disaster rate acceleration to control for recent variation in climate volatility at the local level*.* This variable is calculated as the ratio of the disaster rate over the past 10 years to the overall disaster rate over the last 70 years.

### Analysis

Our preferred specification to estimate the causal effect of a daily temperature record on local climate attitudes is as follows:

2 $${Y}_{i}=\mathrm{\alpha }+{\upbeta }_{R}{R}_{c}+{\upbeta }_{M}{\mathbf{X}}_{m,c}+{\upbeta }_{D}{\mathbf{X}}_{d,i}+{\upvarepsilon }_{s},$$

where our dependent variable ${Y}_{i}$ is the response to the following question: “To the best of your knowledge, how did excessive daytime heat across the United States in 2022 compare with previous years?” on a five-point scale where larger values indicate more severity (see Table 1, Panel A). Our key independent variable ${R}_{c}$ is defined as the number of broken daily heat records in county *c* during Summer 2022 (or broken during the full year in some alternative specifications). In alternative specifications, we replace ${R}_{c}$ with the log of daily records. Additionally, ${\mathbf{X}}_{m,c}$ represents a vector of meteorological controls at the county level including abnormal heat, number of extreme heat days, long-term average temperatures, disaster history, and county latitude and longitude. ${\mathbf{X}}_{d,i}$ is an additional vector of demographic controls elicited in the survey that vary at the individual level. Finally, ${\upvarepsilon }_{s}$ is a normally-distributed error clustered at the state level.

### Causal interpretation of estimates

**Figure 4:**
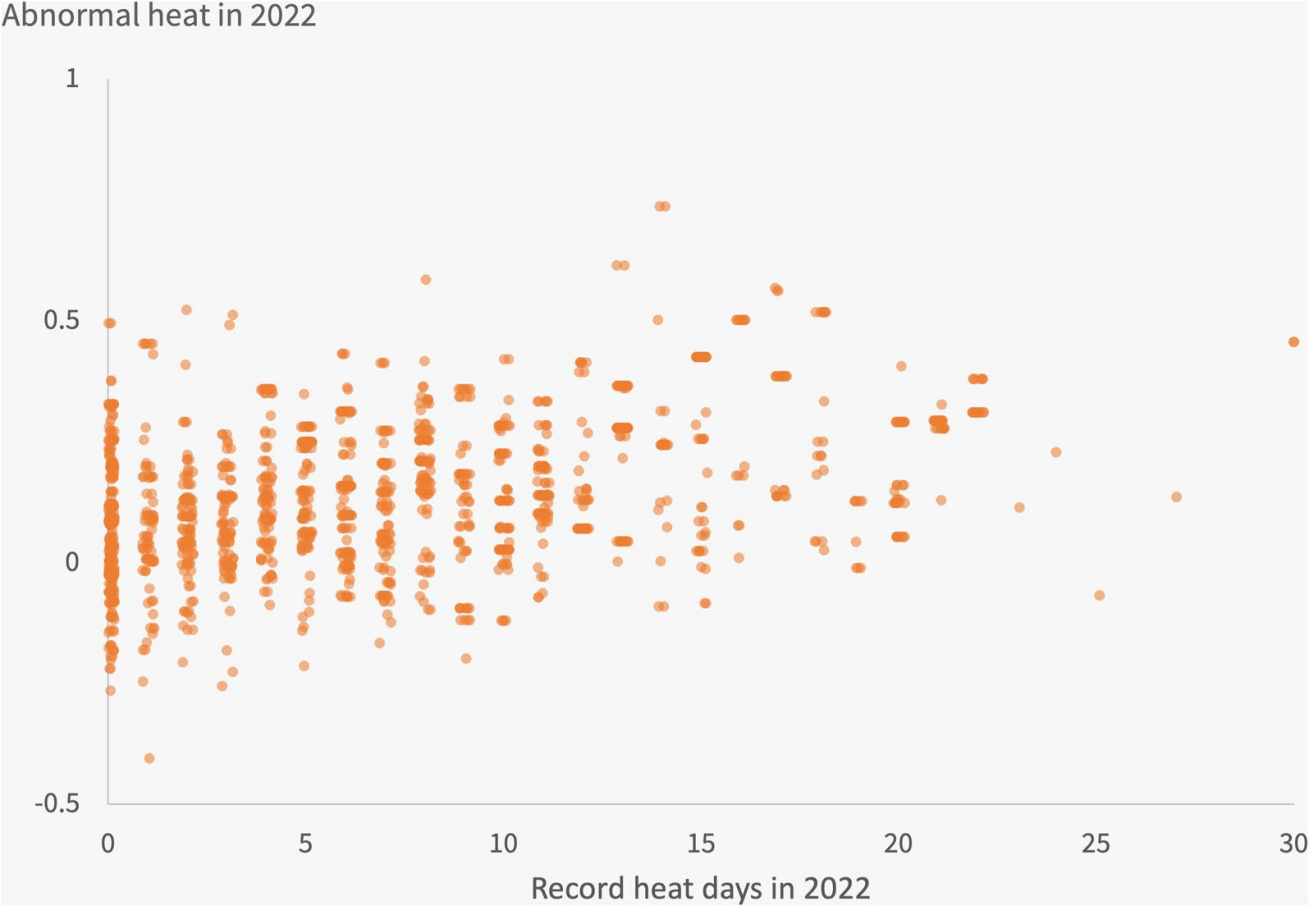
Relation between abnormal heat and record heat days at the county level. A scatterplot of record heat days and abnormal heat. Each point represents one respondent in the sample. Points are jittered horizontally to better visualize the density of data; actual values for the record heat day variable are integers in every case.

Figure 4 illustrates the relatively weak relationship between the number of record heat days (our key independent variable) on the *x*-axis and abnormal heat at the respondent level on the *y*-axis. Recall that abnormal heat is defined as the average deviation of each day’s heat from the historical average for that date, normalized by the standard deviation of temperatures in that month. As expected, there is a statistically significant positive association between these two variables, such that a one-standard deviation increase in abnormal heat (0.15 units) is associated with an additional 2.4 record heat days. Nonetheless, there remains a huge amount of variability in record heat days for a given abnormal heat level. The figure shows many respondents living in counties that had abnormally cold weather in 2022 but many record heat days (bottom right). Likewise, there are many respondents in counties with no record heat days but extremely abnormally hot weather during 2022 (top left). This distribution bolsters our argument that record breaking heat is not closely correlated with underlying county factors, and that differences in our outcome measures across different treatment levels can be attributed causally to record-breaking heat.

### Robustness of results

We use the raw count of record heat days as the dependent variable in most of our analyses. To further test the robustness of the result for heat perceptions, we use different functional forms of the heat record variables and also run ordered logit models that predict responses on the five-point scale without necessarily assuming a linear relation between each point on the scale. Table 6 shows that the results of models run under these various specifications. In each case, the dependent variable remains the same, but the form of the predictor variable and the estimating model varies across columns. The main result is robust to model selection, but statistical significance is lower when the variable is defined in log terms (*p* = 0.07 in Column 2, *p* = 0.06 in Column 4).

**Table 6:** Robustness to alternative model specifications.

	(1)	(2)	(3)	(4)
	OLS models		Ordered logit models	
	Levels	Logs	Levels	Logs
# of record heat days	0.00843** (0.00354)	0.0477* (0.0260)	0.0204** (0.00837)	0.114* (0.0596)
Meteorological controls	✓	✓	✓	✓
Demographic controls	✓	✓	✓	✓
Observations	1605	1605	1605	1605
r2/psuedo R2	0.149	0.149	0.0673	0.0675

OLS and ordered logit regression models where the dependent variable is excessive heat perceptions on a five-point scale. Greater values of the dependent variable indicate belief that excessive heat is more severe in 2022 relative to past years. Columns 1 and 3 use the raw count of record heat days as the main independent variable, while Columns 2 and 4 use the log of the count plus one. Other controls included but not displayed: all those in Table 4, plus disaster rate acceleration, indicators for disasters in 2022, county latitude and longitude, counts of days above 90°F, above 105°F, and above 110°, age band fixed effects, gender fixed effects, education level fixed effects, race fixed effects, county type (metro vs rural) fixed effects, home ownership indicator. The model displayed in Column 1 is identical to that displayed in Table 1, Column 3. Robust standard errors clustered at the state level in parentheses. Statistical significance indicated as follows: ***p < 0.01, **p < 0.05, *p < 0.1

Although we assume that assignment to the record-breaking treatment is equally likely for all counties, assignment is also spatially correlated across counties. If one county experiences a heat wave that leads to records being broken, neighboring counties are also extremely likely to experience high heat and record-breaking weather, as their past weather and record levels will closely resemble the index county. As a result, the assignment of treatment during a given year may leave unbalanced groups that cannot reasonably be treated as counterfactuals for each other. If record-breaking weather just happens to be concentrated in politically conservative areas, for example, this association may lead to a spurious conclusion that record-breaking heat causes counties to become more conservative.

To account for this complication and derive an estimate of the true causal impact of record-breaking temperatures on climate beliefs, our regression models include demographic and meteorological controls to isolate the differences across comparable counties, and we cluster standard errors at the state level to partially account for spatial correlation. We also explicitly account for spatial correlation in a separate regression model to reduce the risk of a spurious finding, and our results are robust to that more generalized model (not shown).

We do not attempt to use a matching procedure to create more comparable treatment and control groups due to the continuous nature of the record days variable. The record heat days variable loses much of its explanatory power if it is dichotomized either at 0 or at the median value.

## Data Availability

Data and replication materials available at https://osf.io/bzjcv/. Sufficient data from the third wave of the longitudinal survey is be provided to replicate the results in combination with the other data sources, all of which are publicly available. The public data is accessible at the following sources: Disaster declaration history: FEMA https://www.fema.gov/openfema-data-page/disaster-declarations-summaries-v1. Weather record history: NOAA https://www.ncdc.noaa.gov/cdo-web/datatools/records. NOAA observation station characteristics: NOAA https://www.ncei.noaa.gov/pub/data/ghcn/daily/ghcnd-stations.txt. Daily temperature history: NOAA https://www.ncei.noaa.gov/data/global-historical-climatology-network-daily/archive/. County centroid latitude and longitude: U.S. Census Bureau https://www.census.gov/geographies/reference-files/time-series/geo/gazetteer-files.html.
